# Diffused responsibility: attributions of responsibility in the use of AI-driven clinical decision support systems

**DOI:** 10.1007/s43681-022-00135-x

**Published:** 2022-01-24

**Authors:** Hannah Bleher, Matthias Braun

**Affiliations:** Friedrich-Alexander University of Erlangen-Nuremberg (FAU), Institute for Systematic Theology, Chair of Systematic Theology II (Ethics), Kochstraße 6, 91054 Erlangen, Germany

**Keywords:** AI, Ethics, Responsibility gap, Decision-making, Human–machine interaction

## Abstract

Good decision-making is a complex endeavor, and particularly so in a health context. The possibilities for day-to-day clinical practice opened up by AI-driven clinical decision support systems (AI-CDSS) give rise to fundamental questions around responsibility. In causal, moral and legal terms the application of AI-CDSS is challenging existing attributions of responsibility. In this context, responsibility gaps are often identified as main problem. Mapping out the changing dynamics and levels of attributing responsibility, we argue in this article that the application of AI-CDSS causes diffusions of responsibility with respect to a causal, moral, and legal dimension. Responsibility diffusion describes the situation where multiple options and several agents can be considered for attributing responsibility. Using the example of an AI-driven ‘digital tumor board’, we illustrate how clinical decision-making is changed and diffusions of responsibility take place. Not denying or attempting to bridge responsibility gaps, we argue that dynamics and ambivalences are inherent in responsibility, which is based on normative considerations such as avoiding experiences of disregard and vulnerability of human life, which are inherently accompanied by a moment of uncertainty, and is characterized by revision openness. Against this background and to avoid responsibility gaps, the article concludes with suggestions for managing responsibility diffusions in clinical decision-making with AI-CDSS.

## Introduction

The task of making good decisions is a complex one, and particularly so in a health context. Clinical decision-making has long benefited from the use of various techniques to support, accelerate or advance decisions. The rise of artificial intelligence (AI) and its implementation in clinical decision-making may significantly impact these practices. Following the definition of the European Commission [1], we define AI in this article as software (and hardware) systems that display intelligent behavior like, for example, making statements and predictions about current or future health conditions, by processing large amounts of data with varying degrees of self-activity. There are numerous examples of AI-driven clinical decision support systems (AI-CDSS) and associated potential benefits to health and wellbeing [2–4]. One such AI-CDSS, for instance, which serves as a key example in this article, is a “digital tumor board” which helps support decision-making on breast cancer treatment by “scoring” the probability of physicians’ decisions [5]. For the field of oncology in particular, but also more generally, such systems promise several benefits to medical decision-making: they process vast amounts of data in a remarkably short time, may boost efficiency in day-to-day clinical operations, and learn adaptively with each data set processed, providing increasingly accurate analysis. Research on AI-CDSS is progressing and has accelerated recently in the context of the COVID-19 pandemic [6–8]. Systematic reviews, however, indicate that such AI-driven predictive or diagnostic models are not yet appropriate for clinical use due to methodological flaws and their susceptibility to bias [9, 10].

The application of AI in the clinical decision-making of the future is likely to challenge established structures of responsibility in this field and, as this article will argue, provoke diffusions of responsibility. The term “diffusion of responsibility” draws on insights from social psychology [11] to denote a phenomenon in which divergent attributions of responsibility to various different agents are possible, or in which attributions of responsibility are manifold, uncertain, or not consolidated in particular administrative, legal or social structures. Such diffusions of responsibility may stem either from the involvement of a large number of parties to whom responsibility may be attributable or from a change in the application context due to, for example, the emergence of new technical capabilities or novel modes or possibilities of human–computer interaction (HCI). Such diffusions may have the potential to disrupt existing attributions or structures of responsibility, leading to a responsibility gap. Instead, they may also lead to redistributions or reorganizations of responsibility, improving possible deficits of structures pertaining to responsibility by giving rise to maybe a fairer arrangement.

This article explores from an ethical perspective how diffusions of responsibility arise, at causal, moral, and legal levels, in clinical contexts involving the use of AI-CDSS. Rather than focusing on bridging responsibility gaps, this article argues that diffusions of responsibility can serve as stimulus for the discovery of new possibilities relating to the attribution of responsibility under changing circumstances, as well as helping prevent the emergence of gaps in the first place. In doing so, we use the example of an AI-driven “digital tumor board” to illustrate changes regarding attributions of responsibility in clinical contexts involving AI-CDSS (“*How a ‘Digital Tumor Board’ Raises Questions of the Attribution of Responsibility*”). The current scientific and public ethical discourse on AI-driven systems proceeds from the assumption that responsibility gaps will become a major problem. While not denying the potential for or problematic nature of responsibility gaps, we propose a shift in perspective toward diffusions of responsibility (“*Diffusions of Responsibility*”), which means, toward a more “resource-oriented” perspective on the changing conditions and possibilities for the attribution of responsibility, as opposed to a deficit-centered focus on the threats and risks associated with responsibility gaps. This line of argument conceives of responsibility as a multidimensional and relational concept, as suggested by normative considerations on the avoidance of inflicting experiences of disregard in light of the vulnerability of human life. We complement this argumentation with a pragmatic ethical approach that emphasizes an inherent moment of uncertainty in responsibility and characterizes the attribution of responsibility as a dynamic endeavor that is open to revision, implying a responsiveness with respect to the contexts, processes and practices of its attribution. We will subsequently discuss three dimensions of the attribution of responsibility, the causal, moral, and legal dimension, enabling us to systematically locate responsibility diffusions (“*Causal, Moral, and Legal Attribution of Responsibility*”). We will also outline in this context how current debates about AI ethics are already addressing these diffusions. Against this backdrop and drawing on the normative considerations we have outlined, the article concludes with suggestions for coping with diffusions of responsibility in clinical settings (“*Managing Diffusions of Responsibility with AI-CDSS in Healthcare*”).

## How a “digital tumor board” raises questions of the attribution of responsibility

The concept of a “digital tumor board” [5] for breast cancer treatment is particularly illustrative of the potential offered by AI-CDSS and the associated challenges of the attribution of responsibility. The traditional process of an “analog” tumor board involves the collection, evaluation, and analysis of data by several medical experts from different disciplines, including, but not limited to, oncologists, radiologists, pathologists, and surgeons, who collaboratively discuss and review cancer patients’ health status and treatment options to the end of determining the best possible treatment plan for each patient. This process involves the provision of comprehensive information to patients about the tumor board’s composition and the matters under discussion. The complexity and time required for data analysis increases with the volume of data available and with the use of novel health-related data sources such as Electronic Health Records (EHR), health apps, and smart health or fitness devices; this increase may go beyond what physicians are able to manage [5].

The use of neural network architecture for data analysis may therefore provide significant support to the tumor board process and the associated decision-making. As Yang et al. [5] explain, the neural network, like the traditional tumor board, evaluates the data with respect to a specific category of potentially suitable treatment—radiation, systemic therapy, or surgery—measuring each treatment type against specific patient data, historical patient data, and the features and values of specific treatments. On the basis of this analysis of data, and trained using historical decisions taken by physicians, the machine learning (ML) model calculates the probability of each decision option and provides recommendation scores on this basis to help physicians make decisions on treatment for patients. The system additionally provides a historical list of similar cases to facilitate comparison. This predictive model can therefore also serve to detect anomalies and support clinicians by searching for similar historical cases [5]. Notwithstanding the limitations of the “digital tumor board” as an example for our purposes—such as a lack of clarity as to how exactly it might work in day-to-day clinical practice and how the system specifically improves clinical decision-making in comparison to the “analog” tumor board—it provides a sound basis for exemplifying the difficulties and opportunities surrounding AI-CDSS now and going forward.

A predictive AI-CDSS based on deep learning differs from other statistical methods by its so-called “intelligence”, or, more accurately, its automatization via machine learning [12, 13]. It models clinical decision-making in a self-learning process with respect to a reference case as a probability-based prediction and provides the user with recommendations. This manner of functioning raises a number of challenges. The use of deep learning to generate predictive statements of probability for a given reference case on the basis of data correlations begs the question of causality: while deep learning algorithms can learn input–output relations in a highly complex manner, the links and intermediaries among variables currently largely elude explication. Even the developers of the algorithms will struggle to explain the interrelationships among the variables or the ultimate outcome. Evidence-based criteria, incorporated into the design of the AI-CDSS as decision parameters, can remedy the causality problem to an extent. However, the technical non-explicability of causality in predictive AI-driven systems remains a challenge—one of particular pertinence to the health context, where the reasons for inferred outcomes may be material to an aspect of a patient’s health, potentially assisting, such as in addressing the causes of a disease or in aiding informed patient consent by disclosing the grounds upon which a decision on treatment has been reached. Causal models of AI, this means models that can identify causal relationships from the data itself, seem to offer a solution with respect to the causality problem, yet are currently difficult to implement on a technical level due to the associated systems’ complexity and a lack of sound and scalable algorithms. However, research is in progress in this area [14–17], which may change this situation in the future.

In addition to these aspects of causality, if AI-CDSS rank possible diagnoses or prognoses or recommend treatments (without disclosing causality), such rankings may alter or at the least influence physicians’ and patients’ decision-making. For example, if highly accurate and reliable systems outperform experts (as in the case of skin cancer [18] and in the detection of diabetic retinopathy [19]), the interaction could change accordingly; it may be, for instance, that the physician trusts herself less and relies more heavily on the support system. Alternatively, the role of the patient may change. There are conceivable future scenarios in which the decision-making process through AI-CDSS takes place in direct interaction with the patient. AI-CDSS such as the “digital tumor board”, however, are currently not capable of making medical assessments by physicians obsolete. Further, their efficiency for public health in general and day-to-day clinical routines in particular, in terms of time and expense, has yet to be sufficiently demonstrated [20]. At best, AI-CDSS may currently be able to serve in a supportive role for consultations—to cite our principal example—of “analog” tumor boards. This said, empirical findings on digital health suggest that competencies, expectations, and interaction relationships in context with digital devices and support systems are changing [21]. Related to this, we expect shifts in normative conceptions with regard to aspects of the decision-making process such as trustworthiness, transparency, agency, and responsibility [22, 23].

For addressing these shifts and embracing the transformational potential of AI-CDSS in regard to responsibility, we want to emphasize three key changes in decision-making effected by these systems, each of which are of particular relevance to the “digital tumor board” example. First, AI-CDSS change decision-making at a factual level, meaning that the actual decision process changes due to emerging technical conditions and new circumstances. They generate automated data analyses by opaque processes of AI systems, so-called “black boxing”, on the basis of correlations and calculations of probability. Here a question emerges, in connection with the epistemological issues around causality, as to the extent to which it is possible to trace responsibility back to specific decision-making processes; or, in other words: Who or what is causally responsible for a particular outcome of treatment? Second, at a societal and individual level, the use of a “digital tumor board” raises the question of who or what is morally responsible when an incorrect treatment recommendation generated by an AI-driven system results in harm to a patient. Alongside the change in modes of interaction and roles brought about by AI-CDSS comes an expansion in the number of possible moral agents involved in a decision: the system’s developers; the providers of technologies, such as software or tech companies; the system’s operators, such as hospitals or other public health institutions; all data subjects who enter or have previously entered their data into the system—all these and others may be deemed agents in the context of AI-CDSS. Third, the difficulty of identifying both causal and moral responsibility is compounded by that of embedding these changes into a regulatory and organizational framework with legal force. The consequences of human–machine interaction in terms of changes in patients’ roles and in their expectations toward physicians are of particular interest here. Is the attending physician, the head physician in a team or department, or the medical director of a clinical setting responsible for the AI-CDSS and its outcomes? Is there a possibility of setting up other structures of responsibility for cases of harm? A system cannot by itself provide monetary compensation for a poor outcome. There is therefore a need for regulatory and organizational structures which can ensure that claims for damages are legally enforceable and do not fizzle out, or in other words, may not be addressable.

**Fig. 1:**
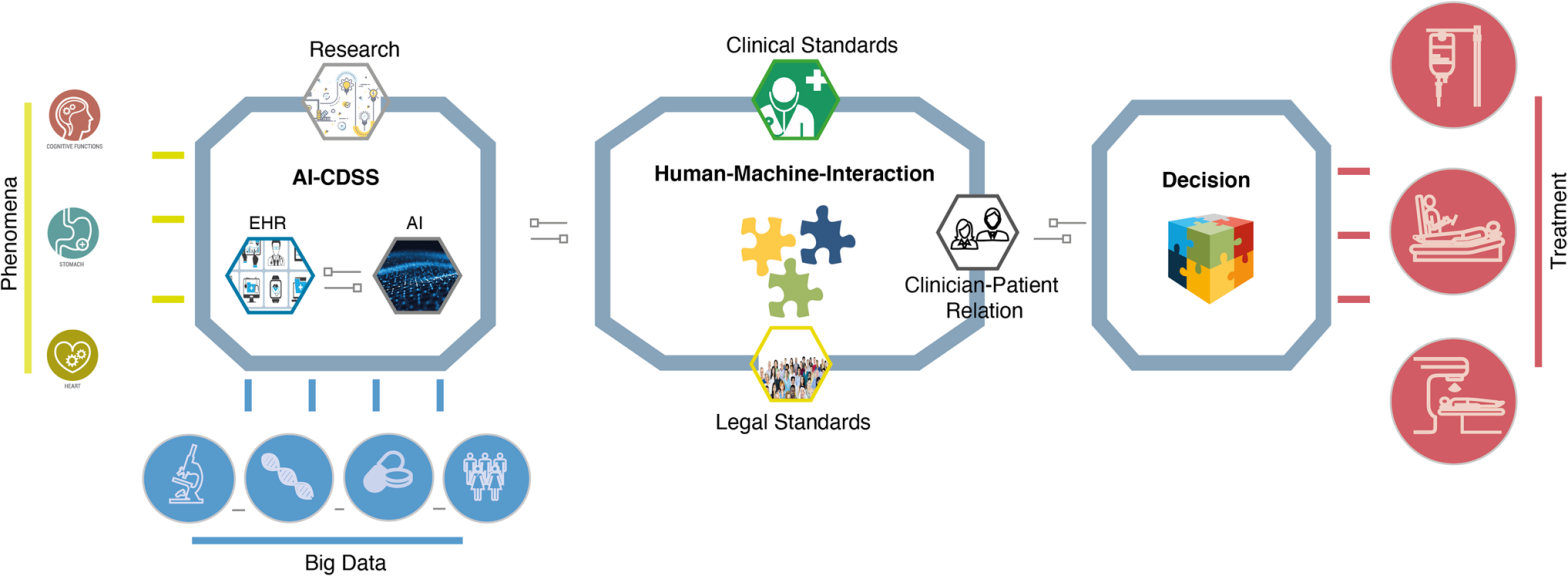
Clinical decision-making with AI-CDSS focuses on the *design* of AI-CDSS and related data generation and data analysis, is characterized by *human–machine-interaction*, and finally aims at the management of the outcome of the *decision* on treatment of patients

As Fig. 1 illustrates, AI-CDSS bring three central issues into focus: the systems’ design, the human–machine interaction, and the outcome of the decision reached, its management and evaluation.

## Diffusions of responsibility

Various voices in current debates on responsibility in the context of AI systems have identified a “responsibility gap”—or a multiplicity of different gaps, as Santoni de Sio and Mecacci [24] propose—as a major problem with the systems’ use [25–30]. The concept of a “responsibility gap” assumes that, as the activities of AI systems become increasingly automated, a point will arrive at which no agent will have enough control to take responsibility or be held responsible for outcomes. Other scholars assert that no such gap exists [31–33] One of them is Daniel Tigard [32], who argues instead for a pluralistic approach to moral responsibility, stating that it is quite usual to attribute responsibility in complicated situations and to unconventional subjects. In light of this, Tigard argues that there is no technology-based gap of responsibility. An illustrative but not technical example in this regard is that it is common to assign responsibility for damage caused by pets—including legal responsibility—to their owners, even if the latter exert no direct control over the actions of the animal and the unforeseeable harm it may cause [31, 32].

This article’s examination of the shifts and transformations in attributions of responsibility with the use of AI-CDSS will focus neither on bridging responsibility gaps nor on refuting their existence. Instead, it argues for an approach revolving around the addressing and consideration of diffusions of responsibility engendered by the use of AI-CDSS. Following in this sense Tigard’s line of argument, engagement with diffusions of responsibility emphasizes the dynamics and complexities inherent in responsibility and regards responsibility as multidimensional and relational. Responsibility depends on various attributions related to a subject, an object, an action and its outcome or consequences, a specific setting, and recipients of responsibility, those for whom responsibility is taken vicariously. We can identify various different forms of responsibility, such as accountability, culpability, and liability, distinguish between attributing, assigning, and assuming responsibility, and differentiate among understandings of responsibility as either an obligation or a virtue of a role, profession, or task [34]. The attribution of responsibility may consequently take place in manifold and complex ways. From this perspective, a deficit-oriented discourse that focuses exclusively on how to bridge a responsibility gap is misguided. Instead, we advocate a resource-centered discourse that places emphasis on the multiple options and possibilities for assigning responsibility in order to avoid a gap arising in the first place. To this end, it is helpful to explore the diffusions of responsibility that may emerge in new contexts and situations, such as the use of technologies like AI-CDSS. In this spirit, a focus on diffusions can do justice to the multidimensionality and relationality of responsibility and shed light on how structures of responsibility come to be and the possibilities they entail.

The term “diffusion of responsibility” does not denote a “new” phenomenon. In social psychology, it describes a situation in which a number of potential attributions of responsibility to various agents are possible. The most common manifestation of the phenomenon is the “bystander effect”, occurring in situations in which no one feels responsible enough to act responsibly [11, 35]. Diffusions also are well-known in other contexts; one instance is the situation in law in which responsibility cannot be assigned to a particular agent because the legal conditions for such attribution of responsibility are not or only partially met [36]. This may occur when obligations or tasks are assigned to a collective entity rather than one particular agent, or difficulties emerge in identifying who has done what because too many agents are involved [36, 37]. A further form of diffusion in the legal context appears in the attributability of different forms of responsibility, such as accountability, culpability, and liability, to different agents for one and the same incident. The clinical context serves as an illustrative example in this regard: While in German hospitals the “head physician” (*Chefärztin*) of a department or specialty is legally responsible for it as its general medical director, the hospital’s management assumes responsibility for its staff and equipment. Depending on the contractual arrangements in place at the hospital and with specific physicians, either a patient’s attending physician, the medical director, the hospital, or all of them may find themselves held responsible for a poor outcome of medical treatment. The attending physician, nevertheless, may be sued personally for harm in any cases of medical malpractice. A number of attributions of responsibility may accumulate in such situations, especially where medical and organizational issues correlate.

Diffusions in this sense, however, carry the potential to cause disruption and responsibility gaps may ensue. This may be the case when, for example, a situation is so complex, unforeseen or risky that it is impossible to attribute responsibility directly to someone or something specific. The response may be to consider a number of agents responsible or to leave the question unresolved, which will mean that no one acts responsibly or dares to take responsibility. In the worst case, the attribution of responsibility is vague, or obscured by complexity, to such an extent that a control or knowledge gap emerges and frustrates any processes of establishing damages and claiming compensation for harm; apathy may take hold, as observed in the “bystander effect” phenomenon; and harm prevails. Responsibility gaps, thus considered, are not inherent to the use of AI-CDSS, nor are they a necessary consequence of this use. They are, however, a risk associated with the diffusion of responsibility. This article makes the case for regarding responsibility gaps as products of mismanaged diffusions of responsibility. As such, our intention is not to deny the existence of responsibility gaps, but rather to prevent them by containing the disruptive potential of diffusion. To this end, we advocate a shift in perspective, which would entail having regard to the diffusion of responsibility and its management rather than seeking solutions to a gap that does not inevitably arise.

## (Re)Thinking responsibility in terms of diffusion

A central lesson from decades of clinical decision-making is that responsibility is not simply an abstract category, but requires definition, justification and specification within practically applied pathways. With this in mind, we propose in this section to supplement our argument by outlining guiding normative concepts of the attribution of responsibility, which underpin our understanding of responsibility as multidimensional and relational. These concepts are the vulnerability of human life as the fundament underlying the notion of responsibility; the uncertainty inherent to the act of taking responsibility, in terms of risk-taking and read in light of the development of a pragmatic ethical theory; and the revisability of responsibility and its attribution, which references the provisional and situational nature of responsibility and the associated capacity for its calling into question and its consequent adjustment.

The first dimension of responsibility we will advance here perceives a multidimensional and, in particular, a relational concept of responsibility as rooted in an understanding of human life as vulnerable [38]. A primary meaning of vulnerability relates to each person’s fundamental experience of the otherness inscribed into their self-reference at the most profound level. In this sense, the vulnerability of the self is not pathological, but rather represents a basic experience encountered in every person’s self-reference. This experience of otherness determines an individual’s self-referentiality as a response to the experience of an Other’s claim or vulnerability. In other words, vulnerability engenders a conception of the self as responsive—a responsiveness not chosen by the individual, but rather, crucially, attributed to her by and with others. This is the moment of transition from responsiveness to responsibility; in the words of the philosopher Emmanuel Lévinas, responsibility describes the individual’s response to the claim of another [39].

Another reading of vulnerability defines it as an individual’s specific experience of another agent’s disregard for their fundamental claims to selfhood. Vulnerability in this sense provides a rationale for responsibility; there would be no need for any assumption of responsibility if there were no call to address the vulnerability of a person or a social group, a process that entails perceiving the specific situation in its social context as a relevant parameter of reflection and, in doing so, allowing all needs and expectations of all agents concerned with the attribution of responsibility to be heard. This type of relational approach exposes the one-sided character of perspectives that encompass either only the person or entity who is responsible, or only the “patient” of that person’s or entity’s act. It diverges from this one-sidedness by considering all stakeholders in their vulnerability, their dependency, and their previous experiences of disregard. In this spirit, an approach focusing on the diffusion of responsibility in terms of avoiding responsibility gaps will seek to avoid the occurrence of new experiences of disregard and injury by establishing appropriate and fair structures of responsibility to the end of enabling rather than preventing free self-determination.

Moving to the second concept, we note that the understanding of responsibility we advance in this article is driven by the assumption that every responsibility is associated with “daring” [40]. Risk assessment and action for risk reduction cannot prevent every occurrence of a damaging event. In principle, every action is associated with unpredictability. The assumption of responsibility is a conscious “daring” to be responsible, accompanied by evaluation and acceptance of the act’s outcome and potential consequences. The notion of “daring” to assume responsibility encompasses the obvious insight that mistakes can be made and that it is possible to be knowingly ignorant of potential consequences or unforeseeable harms when stepping into the breach for someone or something else. The element of “daring” thus indicates that responsibility always remains a risk. John Dewey’s [41] observation that it is not certainty that guides ethical action, but uncertainty, is particularly apposite in this context. Uncertainty, understood here as the driving force of any kind of activity, underlines the character of “daring” that accompanies any action to which responsibility attaches and highlights the importance of the specific situation for the assessment of this action. Guiding criteria for responsible action emerge only in context and through ethical reflection on principles as they operate in that particular situation. Accordingly, this implies that if the situation changes, new diffusions may arise, or, if established structures of responsibility prove unsustainable, a readjustment of responsibility and the criteria used to assess it will need to take place.

The third aspect of responsibility we propose and highlight is its openness to revision, its revisability. The provisional and situational character of responsibility renders it invariably accessible to adjustment and to calling into question. Human vulnerability as a state of responsiveness and the uncertainty which points up the influence of contextual and situational circumstances on responsibility imply such revisability. The temporal dimension of responsibility is a further key element of this characteristic in our context. In AI-driven systems, the point in time at which responsibility is assumed is an essential aspect of the attribution of responsibility [42]: Software development consists in a chronological chain of actions that link various discrete agents. One of the factors upon which the ascription of accountability is contingent is the matter of when someone can be held accountable for something. This temporal dimension also encompasses the insight that every specific assumption of responsibility is time-limited by dint of that specificity Parents, for example, care for their children, but children grow and, in most instances, become able to take responsibility for themselves, at which point the responsibility of the parents changes and new modes of responsibility emerge. A similar point applies to our example of a “digital tumor board” and further future technological opportunities and developments in this context. Future technological advances such as causal models of AI will create significant change in the conditions in which the attribution of responsibility takes place. Once there is a fully automated system which is capable of communicating directly with patients, for instance the frameworks and settings of medical consultations and healthcare practices will shift, as will attributions of responsibility. Attributions of responsibility therefore are fundamental “for the time being” and take place in a specific situation embedded in time and to the best of our knowledge and belief. Any conception of responsibility in this context will need to incorporate its property of revisability and define pathways for the assessment of its attribution.

## Causal, moral and legal attributions of responsibility

In context of this normative approach, we will now explore how diffusions of responsibility unfold in the use of AI-CDSS along three dimensions of the attribution of responsibility. These dimensions, drawn from the three fundamental changes in decision-making described above as impacts of AI-CDSS, are the causal, the moral, and the legal. This outline also highlights and maps current debates in the field of AI ethics across these three levels to illustrate how they address diffusions of responsibility and their causes in the ongoing scientific ethical discourse.

### Causal attribution of responsibility

The endeavor of attributing causal responsibility for events of harm sustained from misdiagnosis or misguided treatment due to the use of AI-CDSS faces the difficulty of locating and tracing errors in AI-driven processes. Questions that may arise include: Did the error occur in the system, in the machine learning processes driven by adaptive learning algorithms? Or was the system trained with a biased data set? Did the error occur during the data collection and acquisition process? Did something go wrong when the system was in use? Or did the user cause the error? Which algorithm, software, mechanisms, or tools caused the error? Even the designers of a system will struggle, in the face of the “black box” processes undertaken by AI-CDSS and on account of the systems adaptive learning processes, with analysis and retracing of the decision made. In the case of a “digital tumor board” that runs through a tremendous amount of data and complex data processes over which it is virtually impossible to exercise knowledge or control, in light if this, it will be challenging to attribute responsibility for the system’s decision or its potential consequences to any specific agent. This leads inevitably to a second pair of questions: What may the object be, for which responsibility can be attributed—the algorithm, the data set, the processing of the data, the system statements, the consequences of decisions arrived at by AI-driven systems? And for which actions or AI-driven system operations may responsibility be attributed?

This “black box” decision-making process imposes severe limitations on control and on the epistemic conditions of knowledge, which, at least in an Aristotelian tradition, are essential to any assumption of responsibility [29, 42–44]. The assumption of responsibility, in this tradition, is indissolubly linked either to the free will to act or to the causation of the act and existence of sufficient control over that act. Further, responsibility in this school of thought depends on relevant knowledge about the decision to be made and its possible consequences. To the end of managing this challenge of controllability and the epistemic uncertainties around “black box” decision-making, recently issued ethical guidelines and codices give prominence to the issue of transparency [45] and thus, in technical, legal, economic, political, and social terms, to the call for the development of explainable AI (XAI). XAI appears to have evolved into a new moral principle for the development and design of responsible AI [46, 47]. Applying this concept to the idea of the “digital tumor board”, it seems clear that explainability is a central aspect of a clinician’s assessment and is particularly key to the justification of a decision to the patient affected. To the end of obtaining informed patient consent to a course of treatment, it seems reasonable to ensure the technical and organizational disclosure of the machine-based and physician-related decision criteria and parameters. Explainability in practice would therefore center the perspective of patients as data subjects by providing them with reasons to contest the decisions if the outcome is not as desired or has caused harm. The provision of access to an understanding of algorithmic decision-making is thus also a basis for action and change as Wachter et al. [48] argue. We might state in this context that explainability attempts to bridge the epistemic and controllability gaps to the end of enabling data subjects to manage their data responsibly.

Notwithstanding the drive to eliminate or at least contain the epistemic and control uncertainties associated with the “black box problem”, it is debatable whether “opening” the black box is a feasible or desirable aim in the medical context. The question arises as to the extent to which, and the instances in which explainability is required, or, looked at the other way around, opacity is acceptable. According to Alex London [49], the demand for explainability of ML may even reproduce the misconception that medical decision-making by physicians is in any way more consistent or explainable than ML-based decision-making. Epistemic questions of explainability and comprehensibility arise with respect to decisions taken by physicians, just as they do with ML; London argues that such decisions are influenced by a mixture of the physician’s experience, associations, and causal evidence. London [49] insists that empirical validation of the reliability and accuracy of ML models should take precedence over their explainability, and asserts that explainability in the sense of interpretability can prove deceptive or harmful in certain situations. If we were to concur with this line of argument in the case of the “digital tumor board”, we might conclude that the matter of how the AI-CDSS reaches its conclusions may not be of interest to clinicians or patients, as long as the system is precise, accurate, takes medical parameters sufficiently into account and is embedded in a comprehensive decision-making process as one component thereof. In line with this thought, it might be worth considering comparing transparency claims and requirements with cases of non-transparency in the same or similar contexts. In other words, we suppose to evaluate the design of transparency, its technical, or social requirements in light of the needs of the relevant parties and contexts. There are areas, especially in the medical context, in which opacity is easily accepted. In this case, most patients, even when provided with medical information, will have little knowledge of or even interest in the exact biochemical workings of a medication. As long as the hoped-for effect occurs, they are likely to accept the risk of side-effects. Analogously, very few clinicians are likely to be well-informed regarding the functioning of and the technical processes underlying software used in the healthcare system. As long as the software fulfills its purpose—such as image recognition in computed tomography—clinicians will typically consider precise knowledge and explainability of data processing procedures to be irrelevant. Expectations that an AI-driven system be explainable require from this perspective assessment of these contextual needs of patients and contexts in coherence with other medically used instruments. All this raises the question of whether there are areas in which “black box” AI processes are acceptable, given appropriate evidence-based reasons? Managing the diffusion of responsibility on a causal level, to contain the epistemic problems, thus requires the discussion of criteria for the explainability of AI-CDSS in relation to its specific context.

At the threshold from causal to moral attribution of responsibility, the implementation of controllability functions and tools to ensure a certain level of control or to avoid failures or malfunctions emerges as an important addition to aspects on the epistemic challenges. Accordingly, alongside considerations around XAI, it will be necessary to take into account the robustness, reliability, and accuracy of the system’s output, in terms of control in the sense of monitoring or oversight of AI-CDSS. Control opportunities are quite relevant when the causal responsibility implies or infers moral attributions of responsibility. Should a “digital tumor board” prove prone to malfunctions and errors or exhibit IT security flaws with respect to patient data, the causal attribution of responsibility will change accordingly; depending on the cause of the malfunctions and who has knowledge of and could be expected to have control over them, responsibility may shift to the developers, providers, or users of the AI-CDSS. The attribution of causal responsibility in this sense crucially depends on robust performance, accurate output, and IT security measures such as the prevention of hacker attacks. The establishment of tools for controllability in the sense of opportunities of monitoring or oversight is an important aspect of maintaining knowledge of and control over the AI-driven system to manage the threshold from causal to moral attribution of responsibility and not letting it become diffuse.

### Moral attribution of responsibility

The implementation of AI-CDSS in the medical context raises the question of the extent of the moral responsibility for the outcomes of their use which may be attributable to clinicians, software and tech companies as providers, computer scientists as developers, or the system itself, or indeed patients or other moral agents. A clinician, relies on the recommendation of the “digital tumor board”, as generated by AI-driven data evaluation, and adjusts a patient’s treatment accordingly. The patient subsequently suffers harm, and the recommendation and the treatment decision that ensued from it turn out to be mistaken. For example, the AI-generated recommendation was for surgery as opposed to radiotherapy, but the individual health status of the patient in question made surgery a risk, and significant harm was the result. At this point, alongside causal questions, moral issues arise as to the extent to which the clinician is morally responsible for the harm sustained, especially if, for instance, they would have made a different decision without the support of the system. It is even imaginable that they had fundamentally disagreed with the system’s decision but had still followed its recommendation, because her previous experience was that the system had always been correct. Is it fair in this situation to hold only the clinician accountable? And what about instances in which, a highly automated system interacts directly with the patient and gives medical recommendations and advice? In this scenario, if harm occurred, would the patient be at fault for following the system’s recommendations, or would the system’s developers, its deployers, or other stakeholders be held responsible?

A key point of discussion in this context is whether moral responsibility can legitimately attach to the system itself. The academic debate has linked this question closely to that of whether an AI system can be considered a “moral agent” [50–52]. The issue of moral agency arises frequently in relation to the automated activities or, as we often hear in the debate, the so-called “autonomy” of AI-driven systems; the term “autonomy” in this context signifies these systems’ use of automated data processing and evaluation mechanisms through which they adaptively, that is, via self-learning algorithms or neural networks, arrive at intelligent, unpredictable evaluations of data. With each set of data they process, they learn and improve their decision-making. These evaluations of data can then lead to system operations and actions (as in robotics) or to data analysis and therefrom to decisions (as in the case of AI-CDSS) on the basis of data correlations and probabilistic calculations. This form of “intelligence” adds pertinence to the debate on the moral agency of robots and AI-driven systems, not least because a key property of this type of machine is its interactivity. Some of these systems are sensitive to their environment, process live data, and can thus respond directly to external inputs, people, animals, things, movements, and situational changes in their surroundings. The view taken on these automated system activities and the system’s ability to interact determines the attribution or negation of moral agency or moral consideration to or in AI-driven systems [26, 50–54]. A prevailing opinion asserts that robots or similar automated systems—among them the “digital tumor board”—cannot be regarded as full moral agents due to (for example) their lack of emotionality, freedom, or awareness of self [42, 53, 55]. Responding to this view, Sven Nyholm explains that, notwithstanding the refutation of full moral agency, it is possible to identify “acceptable ways of interpreting robots as some sorts of agent” [[30], p. 42]. An analogous position emerges in the argument put forward by Behdadi and Munthe [50] as well as by Coeckelbergh [42] that artificial entities, while not full moral agents, are morally relevant to considerations of the attribution of responsibility and definable as “quasi-moral” [53] agents: “We should stop asking questions of what the conditions for being a moral agent are, and whether or not artificial entities may meet those conditions. Instead, we should ask how and to what extent artificial entities should be incorporated into human and social practices that would normally have us ascribe moral agency and responsibility to participants” [[50], p. 32]]. These views appear to indicate the emergence of an overall “relational turn” in debates on the moral status and agency of automated systems [56– 58].

Against this background we argue, that the simple ascription of a particular moral status to an AI-driven system such as the “digital tumor board” does not clarify how we are to attribute responsibility on this basis [42], especially in cases where neither the “humans in the loop” nor the system itself can be held solely responsible for harm sustained. Suppose, for instance, the system processes ran precisely, the users did everything correctly and interpreted the data sensibly, but the data set used caused bias in the decision-making process—perhaps the historical data used by the “digital tumor board” is biased and the data analysis arrived at discriminatory conclusions founded in socio-economic conditions or in relation to specific groups of patients due to their having a rare genetic predisposition or being overweight, of a certain age, female or male (a matter of particular pertinence in relation to treatment decisions for breast cancer with the exemplified “digital tumor board”). Although such specific patient characteristics may enable the drawing up of individualized treatment proposals, there is also a risk that particular groups of people may not receive the most effective treatment for them due to a lack of diversified comparative data and training data sets. Who is to blame if bias has led to damaging correlations, discriminatory conclusions, and ultimately harm to individuals?

Both the issue of moral agency and the difficulty of attributing moral responsibility in instances of bias illustrate the central problem of attribution of responsibility at the moral level, namely that, to use the terminology employed by Coeckelbergh [59], of “many hands” and “many things”—the expansion of the possibilities for attribution of moral responsibility to include more agents and the accompanying proliferation, in light of the relational turn, of “things” requiring moral consideration. In the case of the “digital tumor board”, the problem of “many hands” and “many things” manifests in two ways: First, the arrival of the “digital tumor board” on the decision-making scene expands the setting to encompass a whole range of new stakeholders including developers, providers, and the data subjects of the historical data set used. Second, the “digital tumor board” combines various “things” in terms of technologies and software that are interconnected and require consideration in matters of agency and responsibility, as does the social interaction undertaken with and by these technologies [42]. The attribution of moral responsibility to multiple people and things may accumulate or shift depending on the particular manner of AI-CDSS’ incorporation into the specific medical context and on the social interactions that take place within this setting.

### Legal attribution of responsibility

Where damage has occurred, diffusions of responsibility are particularly problematic from a legal point of view with regard to compensation. The discussion that now follows will refer to European jurisprudence and outline the current state of the European discourse on these matters, which appears amply illustrative, particularly with regard to liability issues, for our purpose of highlighting the legal implications of diffused responsibility. The use of AI-CDSS presents a challenge to frameworks of liability [60] just as it challenges the causal and moral attribution of responsibility. The associated debate has encompassed considerations of whether it is accurate or permissible to regard an AI system such as a robot as an e-person in matters of liability, with the consequence that an “autonomous” machine (whatever that may mean) would receive the status of a legal entity [61–64]. Other discussions have examined whether AI systems call for a more comprehensive definition of product liability to the extent of, for example, obliging manufacturers to stop the system or remove it from circulation if damage or harm occurs. Considerations have also taken place around the potential regulation of user liability in terms of strict liability as in the case of vehicle-driver or pet-owner liability. The Report on Safety and Liability Aspects of AI recently issued by the European Commission’s Expert Group on Liability and New Technologies discusses provisions for covering the risks associated with emerging digital technologies [65, 66]. According to the European Commission’s White Paper on AI, considerations on the development of a predictable and sound framework for legal liability in this area are guided by the view that such a framework requires the capacity to address risks and situations such as “[u]ncertainty as regards the allocation of responsibilities” [67] among different agents. This suggests that there is awareness at the political level of the specific dynamics of responsibility diffusions and their disruptive potential as regards the legal allocation and distribution of responsibility.

The fundamental changes in the roles of the agents involved and the likewise changing relationships among them are likely to be of particular relevance to legal considerations around the application of AI-CDSS in the medical context. There may also be concomitant transformations and modifications in the requirements and claims of stakeholders, especially in the case of harm. Depending on the level of automation, the use of AI-CDSS can differ in modes of interaction between clinicians (including physicians and nursing staff) and the system, clinicians and patients, and patients and system [22, 68]. In this context, significant questions arise that affect the self-images of clinicians and patients in particular and exert an impact on their expectations of one another and the system. Clinicians may find themselves required to consider whether they are to trust their own experience and contextual knowledge or decisions made by the system which contradict their own assessment. Matters of trust and authority also come to the fore, varying in accordance with the particular configuration of clinician/patient/machine interaction at work in each instance. For example, the more autonomously a system acts, the greater the difficulty for the patient in identifying the physician’s part in the treatment process, the extent to which they can rely on the decisions of the system, and which participant is the more reliable. Further, the use of AI-CDSS such as the “digital tumor board” alters tasks and roles: patients may, for instance, co-generate data by using wearables and smart devices that monitor their health status during chemotherapy and supply the “digital tumor board” with data; physicians may delegate repetitive tasks such as recording diagnostic results to intelligent devices that deliver their analyses directly to the tumor board database; and AI-driven systems may take over the analysis of data from clinicians and even, given an appropriate degree of automation, independently conduct patient consultations on cancer treatment options. Changes in clinicians’ and patients’ self-conceptions and role perceptions, shifts in authority associated with altered degrees of faith in clinicians or technologies or with re-adjustments in expectations, and re-allocations of tasks may all call into question established conditions of the attribution of responsibility in causal and moral terms and consequently in legal terms.

The changed expectations associated with these human–machine interaction scenarios are as such indicators of where institutionally embedded clinical decision-making processes are changing. In this respect, they are relevant for considerations of responsibility, in particular from a legal point of view, in order to indicate which diffusions are to be discussed politically and contained legally. It is, after all, the political legislative arena which serves as a space for the negotiation of needs and interests expressed by stakeholders and “patients” of responsibility, with the ultimate aim of striking a balance between the interests of users, developers, and providers of AI-driven systems. In light of the relational turn mentioned above in terms of a moral level of attribution, efforts to find ways of articulating the claims and demands of “patients” as well as “subjects” of responsibility thus comes to the fore in order to make legal attributions tenable and to politically legitimate them.

Admittedly, these considerations cannot answer the question to which extent to which non-human agents can assume legal responsibility, nor can they determine to whom or what we attribute or distribute responsibility. Instead, our thoughts on diffusions of responsibility in a legal sense illustrate the importance of opportunities for the articulation of needs and interests on the part of all agents and “patients” to responsibility, especially in changing settings. It therefore appears imperative to broaden the field of negotiation in this regard. Empirical studies of how established clinical decision-making processes change can, in this sense, indicate which transformations are relevant for legal debates. Considering changing role configurations and modes of interaction thus paves the way for answers to the question of how we can or should attribute responsibility in a legal sense.

## Managing diffusions of responsibility with AI-CDSS in healthcare

**Fig. 2:**
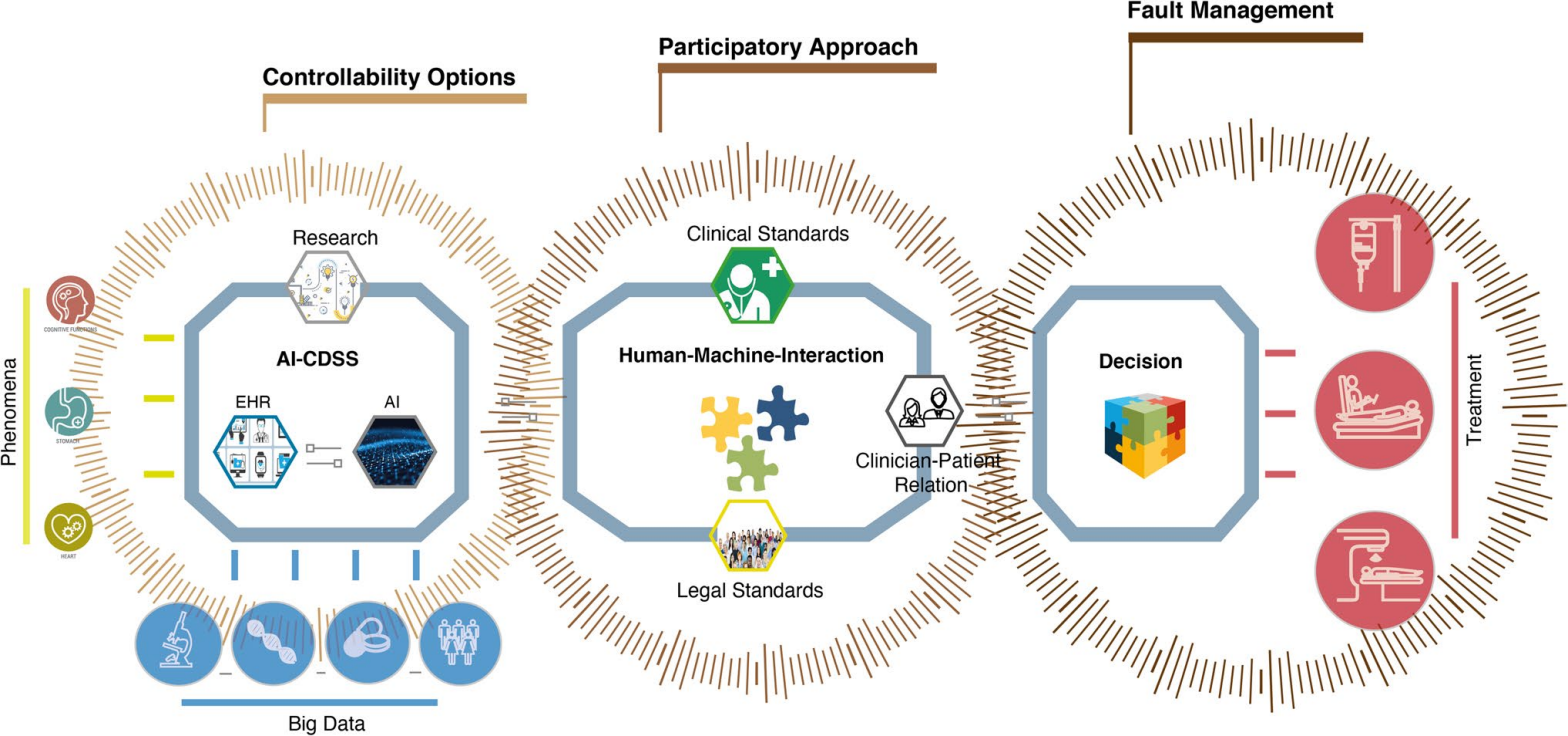
Three factors for addressing responsibility diffusion in clinical decision-making with AI-CDSS: enabling *controllability options* related to the design of AI-CDSS, a *participatory approach* related to human–machine interaction, and establishing *fault management* to complement the management of outcomes of treatment decisions with AI-CDSS

Against this backdrop of diffused or potentially diffused responsibility, and with the issue’s causal, moral, and legal dimensions in mind, the question that remains, in relation to the application of AI-CDSS, is how to manage clinical decision-making and the attribution of responsibility in such a way as to avoid responsibility gaps and safeguard human life in its vulnerability. For this purpose, it appears helpful to focus on the three aspects of the decision-making process involving AI-CDSS (cf. Figure 1): First, the *design of AI-CDSS*; second, the *human–machine interaction* they entail; and third, the *outcome of the decision-making* process for clinical treatment. With regard to these three aspects, we further propose an emphasis on three factors when approaching decision-making and the attribution of responsibility in light of diffusions, via consideration of the normative issues discussed above (vulnerability, uncertainty or “daring”, and revisability): these factors are controllability options, participation, and fault management (cf. Figure 2).

First, the importance of technical design in AI-CDSS has been illustrated by discussing epistemic challenges to responsibility attributions at a causal level, particularly with respect to explainability—but the control aspect of responsibility should not be ignored. We therefore propose an emphasis on the design of *controllability options* for AI-CDSS to the end of enabling defined causal attributions of responsibility. Controllability refers to opportunities for monitoring or control as well as possibilities for engagement by data subjects themselves, such as provided, for instance, in dynamic consent models. Ideally, and in line with the normative considerations outlined above, the design of these controllability options will be processual in character, in terms of its revisability; in other words, there is a need for an abductive approach which will seek to identify when certain options for controllability are required and their necessary extent. Such an approach should take into account the requirements, needs and attitudes of all agents involved as well as the facts and contexts of the associated technical processes, from data generation to data analysis, and likewise the relevant regulatory requirements, moral norms, and causal dependencies. Having regard to the fundamental vulnerability of human life, the guiding principle for such a design process of AI-CDSS should center on avoiding inflicting experiences of disregard like discrimination and violation of personal rights. Effective reflection on all these issues might be most readily accessible with the implementation of an “ethics-by/in/for-design” approach to AI. More generally discursive and deliberative methods of scientific and academic communication, alongside educational and citizen science approaches to AI, will also be important variables for the identification of necessary and useful control mechanisms for the technological implementation of responsible AI design. Such controllability mechanisms will need to be in line with established, tried-and-tested regulations supplemented by a continuous process of review of the standards underlying processes and mechanisms and of their revision via participatory and deliberative procedures in the political sphere and in civil society. This implies that an ethical design process of AI-driven systems will inevitably entail constant and continuous adjustment, which in turn will call for adaptably designed control mechanisms and regulations and sustainable but dynamic risk management.

Second, the clinical application of AI-CDSS places human–machine interaction at the center of the clinical decision-making process. Considering the above-mentioned relational turn and the changes in roles and remits likewise outlined above, these interactions have great potential to induce diffusions of responsibility. We consider it necessary to take a *participatory approach* to the incorporation of AI-CDSS into clinical settings if we are to influence human–machine interaction in its clinical, legal, and social aspects and in its implications for clinical practice; this is particularly imperative for the attainment of sound and predictable legal assessments of responsibility and its attribution. Where the participation of stakeholders is fundamental to the attribution of responsibility, there is space for the articulation of these stakeholders’ expectations, and anyone who has sustained harm can have their complaints heard. In this sense, the deliberation of these demands is particularly relevant and vital for processes of standardization as well as legal considerations on regulations and laws. In this sense, structures for a digital patient participation system could be advanced within the framework of clinical good practice. Those structures, which may be app-based, offer legal, medical but also technical advice as well as information, which could be associated with the use of specific systems such as the “digital tumor board”. It is important that the systems operate in an understandable and instructive way while also including feedback options and consultation services. In addition, patients in particular should be involved as participants in the development of AI-driven technologies from the outset as agents of “ethics-by/in/for-design” approaches to AI in healthcare. Thus, requirements of data subjects and experts can be incorporated into the technology development at an early stage within the framework of collaborative ethical reflection. The incorporation of such participatory elements into the AI-CDSS design process could, in this way, aid critical reflection on attitudes towards the attribution of responsibility early on.

Part of the aim of such a participatory approach to responsibility is to conceptually embed into the design and use of AI-CDSS a continuous process of reflection that opens up the path to revisability. In taking into account all agents involved with their needs and expectations regarding human–machine interaction, we can undertake a deliberative process of consensus-finding around the causal, moral, and legal attribution of responsibility and, above all, respond to the challenges and changes inherent to that process. The importance of this type of engagement emerges particularly strong when we remember that various different forms of individual and, more importantly, collective agency will emerge in each instance depending on the particular configuration of stakeholders involved. The various conceivable forms of collaboration and cooperation will lead to divergent attitudes to responsibility and require different solutions for its distribution. This means that there will not be one single, one-size-fits-all form of attribution and distribution of responsibility which then would be applicable to every case and context of AI-CDSS in use. The attribution of responsibility in relation to databases, for example, is likely to differ from that associated with the robustness or security of systems. The forms of authority and oversight that are desirable, feasible, necessary, and appropriate may thus vary from case to case and will call for specific and conscious clarification in context. In this way, a participatory approach complements a collaborative approach conceptually by including a participatory reflection loop.

Third, in the specific situation of clinical decision-making, considering the potential consequences and outcomes of a decision is a matter of uncertainty or, to put it as we have above, “daring”. If clinical decision-making entails the risk of causing harm and of bearing the associated causal, moral, and legal responsibility, then there is a need to establish sound clinical *fault managemen*t. It is, of course, the case that appropriate risk management, increasing epistemic certainty and security of AI-CDSS, and the application of a precautionary principle all serve to minimize the risk—but it is ultimately the case that failures and errors occur and are to be expected. This potential for error is integral to the management of diffused, and possibly distributed and shared, responsibility.

Clear legal and clinical standards of liability and codes of conduct for the use of AI-CDSS are decisive parameters in appropriate and effective fault management. Nevertheless, it is also important to be aware of the possibility that human–machine interaction might alter the anticipation of errors at a general level. It is likely, depending on the level of trust people invest in the machine and their possibilities for engagement with it, that perceptions of both machine and human error might vary. The further AI-CDSS advance into everyday life, the more likely it is that perceptions and evaluations of error, particularly human error, will change. In this context, it will be crucial to explore the extent to which AI-driven systems are allowed to make mistakes, but also the extent to which people are granted the right to make mistakes themselves, especially when they refrain from using systems or operate them incorrectly, and the subsequent evaluation of these errors. It appears important that fault management in the context of AI-CDSS considers these potential social consequences of the interaction to avoid in a general sense injustice or disregard of individual patients.

Appropriate mechanisms for the management of complaints in the clinical context will be a decisive step in this process, but will require the addition of “spaces” of complaint, compassion, and recognition of harm sustained, spaces whose function will be to enable the clarification of the cause of damage or harm, legal proceedings, and/or allocation of liability, but also forgiveness of mistakes and reconciliation in general. Alongside these social spaces of complaint and redress, we will need spaces of recognition of the other’s vulnerability and fragility. These would represent an extension of clinical feedback opportunities and complaints management and would require sensitive structures of support and care for patients and clinicians alike. A comprehensive information, consultation and feedback service for patients, but also clinicians, would then have to be established when using a “digital tumor board”. It is only on this basis that we can hope to develop solidarity-based solutions for error management such as “risk pooling” [69] and similar models of the structural and financial sharing of blame and liability in accordance with the degree of risk in each specific instance.

With these three aspects we try to set a starting point to tackle the responsibility diffusions on the causal, moral and legal level. At the causal level, responsibility is not only challenged by epistemic issues, but also by a lack of control. Therefore, we advocate for controllability options which are, for instance, already enabled within dynamic consent models. With regard to the moral level, it is necessary to ensure that all respective agents, especially patients, are integrated in AI-based decision-making and AI development through a participatory approach. Offering and implementing concrete pathways and possibilities for participation does not only open up spaces to articulate the respective claims and demands of different agents but is subsequently also an essential prerequisite for the legal attribution of responsibility. At a legal level, diffusions of responsibility are primarily characterized by changes of expectations and perceptions of all the agents concerned in the human–machine interactions. These changes point the path to where and how established decision-making processes change and legal regulations are to be found. One example of this is the different interpretation of human and technical errors. We suggest taking such changes seriously and installing sound clinical fault managements, which in turn provide the basis for solidarity-based instruments like risk pooling. Such fault management, furthermore, captures the risk-taking nature of responsibility associated with a transformative environment by structurally accommodating errors. Exploring the changes induced by AI-driven technologies in the health context and accompanying (new) options for responsibility attributions thus provides, in our opinion, a resource-oriented approach to managing responsibility diffusions at each of the three levels.

## Conclusion

Following the example of a “digital tumor board”, this article explores the changes and shifts of responsibility in using AI-CDSS in the clinical context, at causal, moral, and legal levels. We argue on basis of this description for a shift in perspective towards diffusions of responsibility. This analysis, however, does not deny responsibility gaps but argue to prevent them by focusing on the possibilities for responsibility attributions. For the reason of a more resource-oriented analysis, the article mapped recent discussions and suggestions in the field of AI ethics on responsible AI with the description of diffusions of responsibility at the three different levels. Against this background and based on the understanding of responsibility founded on human vulnerability, we argued for a multidimensional and relational concept of responsibility that we complemented with a pragmatic ethical approach that emphasizes an inherent moment of uncertainty in responsibility and characterizes the attribution of responsibility as a dynamic endeavor that is open to revision. From this stance, three suggestions are made on how to manage responsibility diffusions: first by creating opportunities for control, second by striving for a participatory approach, and third by enabling reliable fault management that takes into account both technical and social aspects of “spaces” of complaint, compassion, and recognition.
